# Evolution of Aroma Profiles in *Vitis vinifera* L. Marselan and Merlot from Grapes to Wines and Difference between Varieties

**DOI:** 10.3390/molecules29143250

**Published:** 2024-07-09

**Authors:** Yi-Lin Ge, Nong-Yu Xia, Ya-Chen Wang, Hua-Lin Zhang, Wei-Ming Yang, Chang-Qing Duan, Qiu-Hong Pan

**Affiliations:** 1 Center for Viticulture and Enology, College of Food Science and Nutritional Engineering, China Agricultural University, Beijing 100083, China; 2 Key Laboratory of Viticulture and Enology, Ministry of Agriculture and Rural Affairs, Beijing 100083, China; 3 Chateau Yuanshi, Yinchuan 750026, Ningxia, China

**Keywords:** dry red wines, aroma profiles, cold soak, wine fermentation, bottle storage

## Abstract

The fermentation process has a significant impact on the aromatic profile of wines, particularly in relation to the difference in fermentation matrix caused by grape varieties. This study investigates the leaching and evolution patterns of aroma compounds in *Vitis vinifera* L. Marselan and Merlot during an industrial-scale vinification process, including the stages of cold soak, alcohol fermentation, malolactic fermentation, and one-year bottle storage. The emphasis is on the differences between the two varieties. The results indicated that most alcohols were rapidly leached during the cold soak stage. Certain C6 alcohols, terpenes, and norisoprenoids showed faster leaching rates in ‘Marselan’, compared to ‘Merlot’. Some branched chain fatty-acid esters, such as ethyl 3-methylbutyrate, ethyl 2-methylbutyrate, and ethyl lactate, consistently increased during the fermentation and bottling stages, with faster accumulation observed in ‘Marselan’. The study combines the Orthogonal Partial Least Squares-Discriminant Analysis (OPLS-DA) model based on odor activity values to elucidate the accumulation of these ethyl esters during bottle storage, compensating for the reduction in fruity aroma resulting from decreased levels of (*E*)-*β*-damascenone. The ‘Marselan’ wine exhibited a more pronounced floral aroma due to its higher level of linalool, compared to the ‘Merlot’ wine. The study unveils the distinctive variation patterns of aroma compounds from grapes to wine across grape varieties. This provides a theoretical framework for the precise regulation of wine aroma and flavor, and holds significant production value.

## 1. Introduction

Aroma is a crucial quality indicator in wine [1]. Currently, over 1300 volatile compounds have been identified in wine, including alcohols, esters, aldehydes, ketones, acids, terpenes, norisoprenoids and sulfur-containing compounds, with concentrations ranging from μg/L to mg/L [2,3,4]. These compounds are typically categorized as varietal aroma, fermentation aroma and aging aroma based on their respective sources [5]. Varietal aroma in wine is primarily contributed by grape berry-derived aroma compounds, such as terpenes, and norisoprenoids, etc., [6]. These compounds play a crucial role in determining the typicality of wine in terms of its variety and region. Their composition and concentration are influenced by both the genotype and the grape-growing environment. Fermentation-derived aroma compounds include alcohols, esters, aldehydes and volatile fatty acids, which are produced by yeast during alcohol fermentation and by lactic acid bacteria during malolactic fermentation, using flavor precursors from grape berries as substrates [7,8]. Therefore, aroma compound profile in grape berries plays an important role in the formation of wine aroma quality.

Aroma compounds exist mainly in grape skins with both free and glycosidically bound forms. The leaching of these compounds and their precursors from grapes and their subsequent conversion in fermentation broth are significantly affected by the cold soak process before fermentation, as well as various microorganisms and grape must components during fermentation [9,10,11,12,13]. It is important to note that per-fermentation cold soak is an aqueous system. Previous studies have primarily focused on the effects of cold soak technology on the color appearance and phenolic compounds of wine [14,15], with little attention given to aroma compounds.

According to research, cold soaking can destroy the cell membrane by increasing liquid volume in grape cells, which leaches aroma compounds [16]. This process can improve the richness of the original fruity aroma of wine and enhance the wine body [17]. Many reports have focused on the effects of fermentation technology on wine aroma production. Recent studies have shown that the interaction between phenolic compounds, polysaccharides, and aroma compounds in fermentation broth has garnered significant attention. It has been found that polyphenols and polysaccharides can influence the volatility of aroma compounds, such as terpenes and esters, through intermolecular interaction forces like hydrogen bonding, dispersion force, and π-π stacking [13,18]. Furthermore, the presence of ethanol in the wine matrix can alter the conformation of proteins, ultimately affecting the binding of protein and aroma molecules [4]. Overall, the release and retention of aroma are influenced by the wine matrix to varying degrees. However, the impact of the matrix on the extraction, conversion, and generation of new aroma compounds during the cold soak and fermentation stages remains unclear. Understanding the similarities and differences in the leaching and conversion of aroma compounds between different varieties is helpful in guiding the production of high-quality wine.

To date, most studies on the evolution of aroma compounds during fermentation and aging have focused on specific winemaking phases rather than the entire process. Additionally, studies have only concerned free aroma compounds that have direct sensory contribution to wine. During fermentation and aging, glycosidically bound aroma compounds from grapes can be hydrolyzed into the corresponding free-form glycones. This process has the potential to significantly contribute to the aroma quality of the final wine. However, there is limited understanding of the changes that occur to these bound compounds during wine fermentation, particularly at an industrial scale. Therefore, investigating the evolution patterns in aroma profiles from grape berries to wine and the influence of varietal differences under industrial scale production conditions is significant. This will clarify the directional regulation of aroma compounds and guide the production of high-quality wines.

The Ningxia region in western China has emerged as a significant wine production area with immense growth potential. Of the red wine grape varieties grown in this region, *Vitis vinifera* L. Merlot has the second-largest planting area, covering approximately 14,500 acres, which is characterized by intense flavors of drupe and tropical fruits [19]. *Vitis vinifera* L. Marselan, a hybrid of ‘Cabernet Sauvignon’ and ‘Black Grenache’, was first introduced to China in 2001. The ‘Marselan’ variety is grown in an area of approximately 1500 acres in the eastern foothills of Helan Mountain. It has a high level of norisoprenoids and a relatively intense floral and fruity aroma, making it a successful crop in many regions of China. In recent years, the planting area of ‘Marselan’ has consistently increased. Compared to ‘Merlot’, the ‘Marselan’ grape has a thicker-skin, darker color, and produces wines with more blackberry, green pepper, honey, raspberry, caramel, smoky, and cinnamon-like aromas [20]. Our recent research shows that the flavonoid concentration is significantly higher in ‘Marselan’ grapes than that in ‘Merlot’, and there are certain differences in the leaching and conversion of phenolic compounds between these two varieties during the winemaking [21]. This study aims to determine whether the difference also occurs in aroma profiling.

The materials used is this study were ‘Marselan’ and ‘Merlot’ grapes from the vineyards located at Chateau Yuanshi of the eastern foothills of Ningxia Helan Mountain. The changes in free- and glycosidically bound aroma compounds in grape juice and wines were investigated during the whole process of cold soak, alcohol fermentation, malolactic fermentation, and one-year bottle storage under the industrial scale production conditions. The aim of this research is to provide guidance for the directional regulation of aroma compounds in the production of dry red wine.

## 2. Results

### 2.1. Aroma Profile of the Ripe Berries of Two Varieties of Grapes

The aroma compounds detected in the ‘Marselan’ and ‘Merlot’ grape berries were categorized into six groups based on their biosynthetic pathways: C6/C9 compounds, aromatic compounds, straight-chain aliphatics, branched-chain aliphatics, terpenes and norisoprenoids. A total of 42 free-form and 23 glycosidically bound aroma compounds were quantified, as presented in Table 1 and Table 2.

#### 2.1.1. Free-Form Aroma Compounds in Two Varieties

Table 1 displays the concentrations of free-form aroma compounds in commercially harvested ‘Marselan’ and ‘Merlot’ grapes. Out of the 42 components, 22 aroma compounds showed higher concentrations in the ‘Marselan’ grapes compared to the ‘Merlot’. These compounds include seven C6/C9 compounds, three straight-chain aliphatics, four aromatic compounds, six terpenes, and two norisoprenoids. Notably, the concentrations of the majority of C6/C9 compounds in the ‘Marselan’ grapes were approximately 2–3 times higher than those in the ‘Merlot’. In particular, (*E*)-2-hexen-1-ol and (*Z*)-3-hexen-1-ol reached levels that were approximately about 5 times and 63 times higher, respectively. Although C6/C9 compounds, also known as green leaf volatiles (GLVs), have a typical “grass flavor”, “green leaf flavor” and other characteristics [13,22], their direct contribution to red wine aromas is limited. This is because they can be rapidly conversed during fermentation and have high sensory thresholds.

**Table 1:** Comparison of free-form aroma compounds between ‘Marselan’ and ‘Merlot’ grapes.

Aroma Components	Retention Index	Standards	Quantitative Methods	Concentrations (μg/kg)	
				Marselan	Merlot
C6/C9 compounds					
1-Hexanal	1090.2	Hexanal	A.Q.	6271.59 ± 821.86 **	3793.55 ± 302.11
3-Hexenal	1142.1	Hexanal	R.Q.	439.67 ± 51.74 **	191.92 ± 35.84
(*E*)-2-Hexenal	1227.4	(*E*)-2-Hexenal	A.Q.	8507.44 ± 929.72 **	4190.59 ± 316.91
1-Hexen-3-ol	1247.3	(*Z*)-3-Hexenol	R.Q.	21.85 ± 3.12	13.12 ± 8.58
1-Hexanol	1349.2	1-Hexanol	A.Q.	392.91 ± 22.53 *	138.11 ± 21.36
(*Z*)-3-Hexen-1-ol	1381.7	(*Z*)-3-Hexenol	A.Q.	592.3 ± 49.64 **	9.28 ± 1.54
1-Nonanal	1397.9	Nonanal	A.Q.	2.64 ± 0.39	2.01 ± 0.46
(*E*)-2-Hexen-1-ol	1402.4	(*E*)-2-Hexenol	A.Q.	1031.45 ± 288.38 **	298.84 ± 42.68
(*E,E*)-2,4-Hexadienal	1410.5	(*E,Z*)-2,6-Nonadienal	R.Q.	53.54 ± 6.01 **	30.33 ± 2.61
(*E*)-2-Nonenal	1543.1	(*E*)-2-Nonenal	A.Q.	0.30 ± 0.05	0.29 ± 0.01
(*E,Z*)-2,6-Nonadienal	1594.8	(*E,Z*)-2,6-Nonadienal	A.Q.	1.17 ± 0.23	1.08 ± 0.15
1-Nonanol	1659.3	2-Nonanol	R.Q.	0.25 ± 0.03	0.22 ± 0.05
Straight-chain aliphatics					
1-Octanal	1005	Octanal	A.Q.	0.37 ± 0.1	0.26 ± 0.14
4-Hexen-1-ol acetate	1321	(*Z*)-3-Hexenyl acetate	R.Q.	3.29 ± 0.35	nd
(*Z*)-2-Penten-1-ol	1317.1	(*Z*)-2-Hexen-1-ol	R.Q.	10.67 ± 0.59 *	6.77 ± 1.79
(*Z*)-2-Heptenal	1190.6	(*Z*)-2-Heptenal	A.Q.	0.75 ± 0.19*	0.35 ± 0.07
(*E*)-2-Octenal	1436.0	(*E*)-2-Heptenal	R.Q.	489,747.86 ± 36,722.12	411,913.44 ± 60,763.77
1-Octen-3-ol	1446.8	1-Octen-3-ol	A.Q.	0.75 ± 0.07	0.65 ± 0.47
(*E,E*)-2,4-Heptadienal	1501.0	(*E,Z*)-2,6-Nonadienal	R.Q.	2.05 ± 0.06 **	1.34 ± 0.18
1-Decanal	1504.1	Decanal	A.Q.	0.45 ± 0.03	0.33 ± 0.1
1-Octanol	1555.4	1-Octanol	A.Q.	0.40 ± 0.04	0.27 ± 0.14
(*E*)-2-Decenoic acid	2271	Decanoic acid	R.Q.	0.34 ± 0.08	0.25 ± 0.02
Aromatic compounds					
p-Cymene	1278.1	p-Cymene	A.Q.	3.63 ± 0.17 **	0.61 ± 0.12
Benzaldehyde	1535.2	Benzaldehyde	A.Q.	24.31 ± 5.27 *	7.12 ± 5.92
Phenylacetaldehyde	1655.8	Phenylacetaldehyde	A.Q.	120.39 ± 22.98 *	43.17 ± 19.87
Acetophenone	1664.4	Phenylacetaldehyde	R.Q.	7.45 ± 1.02	7.13 ± 1.22
Methyl salicylate	1793.3	Methyl salicylate	A.Q.	0.99 ± 0.11	1.20 ± 0.04 *
Phenethyl alcohol	1921.3	Phenethyl alcohol	A.Q.	56.33 ± 1.64 **	11.59 ± 3.45
Phenol	2015.8	Phenol	A.Q.	7.18 ± 0.62	6.08 ± 0.47
Branched-chain aliphatics					
2-Ethylhexanol	1486.6	2-Ethylhexanol	A.Q.	0.87 ± 0.15	0.77 ± 0.15
Terpenes					
*α*-Terpinene	1702.2	Terpinolene	R.Q.	4.25 ± 0.24 **	nd
*γ*-Terpinene	1238.5	Terpinolene	R.Q.	3.48 ± 0.31 **	nd
Terpinolene	1289.9	Terpinolene	A.Q.	2.72 ± 0.09 **	nd
*α*-Terpineol	1702.3	α-Terpineol	A.Q.	1.94 ± 0.37 **	0.30 ± 0.1
Terpinen-4-ol	1608.2	α-Terpineol	R.Q.	1.07 ± 0.12 **	0.29 ± 0.09
Linalool	1546.1	Linalool	A.Q.	4.10 ± 0.49 *	0.36 ± 0.07
L-Menthol	1643.1	α-Terpineol	R.Q.	1.68 ± 0.18	1.65 ± 0.03
Norisoprenoids					
(*E*)-*β*-Damascenone	1835.3	*β*-Damascenone	A.Q.	32.07 ± 8.64 *	10.40 ± 1.97
*α*-Ionene	1541.7	β-Ionone	R.Q.	1.39 ± 0.13 **	nd

Note: * *p* < 0.05; ** *p* < 0.01. nd: not detected; A.Q.: absolute quantification; R.Q.: relative quantification.

Terpenes and norisoprenoids exhibit significant varietal differences, with a greater variety of free-form components found in ‘Marselan’. Furthermore, ‘Marselan’ grapes were found to contain *α*-terpinene, *γ*-terpinene, terpinolene, and *α*-ionene, which were not detected in ‘Merlot’. With the exception of L-menthol, the levels of aroma compounds detected in both varieties were significantly higher in ‘Marselan’ grapes. Notably, (*E*)-*β*-damascenone was the most abundant isoprenoid-derived aroma compound in both varieties, with a concentration in ‘Marselan’ approximately three times higher than that in ‘Merlot’. Grape-derived terpenes and norisoprenoids significantly contribute to the varietal typicality of wine, providing floral and citrus, tropical fruit and violet aromas, respectively [3]. The difference in terpenes and norisoprenoids between ‘Marselan’ and ‘Merlot’ grapes may potentially affect aroma characteristics of their respective wines.

#### 2.1.2. Glycosidically Bound Aroma Compounds in Two Varieties

Table 2 shows 23 glycosidically bound aroma compounds found in the two varieties, comprising six C6/C9 compounds, six straight-chain aliphatics, two branched-chain aliphatics, four aromatic compounds, two terpenes, and three norisoprenoids. Of them, eight components had higher concentrations in ‘Marselan’ grapes, and only (*E*)-2-hexen-1-ol and benzyl alcohol had higher levels in ‘Merlot’. Additionally, as for terpenes and norisoprenoids, bound-form linalool, *α*-terpineol, vitispirane A, and vitispirane B showed significantly higher concentrations in ‘Marselan’ grapes compared to ‘Merlot’. However, no significant difference in the concentration of glycosidically bound (*E*)-*β*-damascenone was observed between the two varieties.

**Table 2:** Comparison of glycosidically bound aroma compounds between ‘Marselan’ and ‘Merlot’ grapes.

Aroma Components	Retention Index	Standards	Quantitative Methods	Concentrations (μg/kg)	
				Marselan	Merlot
C6/C9 compounds					
1-Hexanol	1349.2	1-Hexanol	A.Q.	63.7 ± 6.01 **	40.07 ± 5.15
*(Z)*-3-Hexen-1-ol	1381.7	(*Z*)-3-Hexenol	A.Q.	38.76 ± 4.49 **	11.08 ± 0.88
*(E)*-2-Hexen-1-ol	1402.4	(*E*)-2-Hexenol	A.Q.	44.27 ± 6.54	92.76 ± 3.52 **
2-Nonanol	1084.2	2-Nonanol	A.Q.	0.27 ± 0.02	0.28 ± 0.03
1-Nonanol	1659.3	2-Nonanol	R.Q.	0.32 ± 0.07	0.32 ± 0.13
*(Z)*-3-Nonen-1-ol	1685.6	2-Nonanol	R.Q.	0.17 ± 0.03	0.20 ± 0.01
Straight-chain aliphatics					
2-Heptanol	1315	2-Heptanol	A.Q.	0.66 ± 0.09 **	0.24 ± 0.02
2-Octanol	1550.6	1-Octanol	R.Q.	0.26 ± 0.02	0.28 ± 0.03
1-Octen-3-ol	1446.8	1-Octen-3-ol	A.Q.	0.57 ± 0.09	1.24 ± 0.41
1-Heptanol	1451.5	1-Heptanol	A.Q.	0.68 ± 0.07	0.64 ± 0.2
1-Decanol	1970.2	1-Decanol	A.Q.	0.18 ± 0.05	0.19 ± 0.06
1-Octanol	1555.4	1-Octanol	A.Q.	0.58 ± 0.06	0.62 ± 0.22
Branched-chain aliphatics					
Isoamyl alcohol	1199.8	3-Methylpentanol	R.Q.	7.91 ± 1.42	12.71 ± 5.75
2-Ethylhexanol	1486.6	2-Ethylhexanol	A.Q.	0.52 ± 0.19	0.57 ± 0.11
Aromatic compounds					
Methyl salicylate	1793.3	Methyl salicylate	A.Q.	1.85 ± 0.29	2.92 ± 1.43
Benzyl alcohol	1885.7	Benzyl alcohol	A.Q.	235.87 ± 28.17	512.25 ± 10.05 **
Phenethyl alcohol	1921.3	Phenethyl alcohol	A.Q.	181.72 ± 25.1 *	106.52 ± 21.13
Phenol	2015.8	Phenol	A.Q.	4.50 ± 0.3	4.99 ± 0.37
Terpenes					
Linalool	1546.1	Linalool	A.Q.	1.59 ± 0.19 *	0.76 ± 0.35
*α*-Terpineol	1702.3	α-Terpineol	A.Q.	1.42 ± 0.16 **	0.60 ± 0.11
Norisoprenoids					
Vitispirane A	1539.3	*β*-Damascenone	R.Q.	14.93 ± 1.58 **	8.15 ± 0.09
Vitispirane B	1542	*β*-Damascenone	R.Q.	13.82 ± 1.37 **	7.93 ± 0.39
(*E*)-*β*-Damascenone	1835.3	*β*-Damascenone	A.Q.	20.98 ± 1.6	19.11 ± 0.53

Note: * *p* < 0.05; ** *p* < 0.01. A.Q.: absolute quantification; R.Q.: relative quantification.

### 2.2. Evolution of Aroma Compounds during Cold Maceration Stage

**Figure 1:**
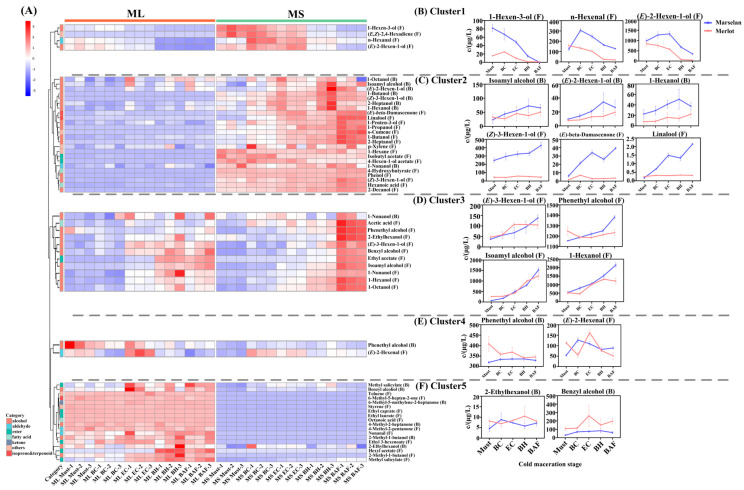
Changes in free−form and glycosidically bound aroma compounds during the cold soak stage. (**A**) clustered heat map analysis; (**B**–**F**): aroma compounds with typical variation trend in each cluster. In the heat map, the letter F after the compound name represents the free form, and the letter B represents the bound form.

During the pre-fermentation cold soak stage, the grape mash is in an aqueous system. To better understand the leaching patterns of aroma compounds, both free-form and glycosidically bound, sampling was performed at five different time points during this stage. An analysis of clustering heat map was used to explore the changes in compounds. A total of 43 free and 16 bound aroma compounds were leached from “Marselan” and “Merlot” grapes during this stage. The compounds were clustered into five groups based on their concentration variations in the two varieties, as illustrated in Figure 1. The details of concentrations of substances can be found in Table S1 (Supplementary Materials).

Aroma compounds in cluster 1 showed a continuous decrease in concentration during the cold soak stage, with the concentration approaching zero before alcohol fermentation (BAF). The results presented in Figure 1B show that free-form 1-hexen-3-ol, 1-hexanal, 1-hexanal, and (*E*)-2-hexen-1-ol experienced the greatest decrease. This suggests that these C6 compounds can be rapidly extracted from grape berries and subsequently transformed into other components after leaching.

The compounds in the cluster 2 and cluster 3 showed an increasing trend throughout the soaking. Cluster 2 was composed of seven bound-form alcohols, six free-form alcohols, as well as free-form (*E*)-*β*-damascenone and linalool. In this cluster, aroma compounds were continuously released, and their concentration varied significantly between the varieties, especially for free-form compounds. Notably, the concentrations of linalool and (*E*)-*β*-damascenone in ‘Marselan’ showed a clear upward trend, while they remained at a low level in ‘Merlot’ (Figure 1C). However, the levels of glycosidically bound alcohols, isoamyl alcohol, (*E*)-2-hexen-1-ol, and 1-hexanol showed a continuous increase in both ‘Marselan’ and ‘Merlot’. ‘Marselan’ had a higher overall increasing rate compared to ‘Merlot’ (Figure 1C). Cluster 3 contained 11 aroma compounds, most of which were in free form, with the exception of ethyl acetate and 1-nonanol in the bound form. Additionally, these compounds in Cluster 3 did not show significant difference between two varieties, with similar leaching rates (Figure 1D).

The compounds in cluster 4 and cluster 5 exhibited minimal fluctuations during the soaking process. Cluster 4 primarily comprised of bound-form phenethyl alcohol and free-form (*E*)-2-hexenal, while cluster 5 mainly consisted of aromatic alcohols and ketones. It is worth noting that most aroma compounds were only detected in ‘Merlot’.

### 2.3. Evolution of Aroma Compounds during Fermentation and Bottle Storage

Unlike the cold soak stage, the leaching and evolution of aroma compounds occur in the alcohol phase during the fermentation and bottle stages. In the early stages of alcoholic fermentation, a large number of esters, including a variety of ethyl esters and acetates, and a variety of alcohols are produced (Figure S1). A total of 59 free-form and 18 bound-form aroma compounds were identified in ‘Merlot’ and ‘Marselan’ during the fermentation stage. The details of concentrations of compounds can be found in Table S2. Free-form ethyl phenylacetate, isobutyl octanoate, (*Z*)-3-hexene-1-ol, 1-pentanol, butanoic acid, octanoic acid, and p-cymene were detected only in ‘Marselan’, along with bound-form 1-butanol and isobutanoic acid. Conversely, free-form ethyl valerate, ethyl lactate, and decanoic acid, as well as bound-form 4-methyl-2-heptanol and 2-methyl-1-butanol, were identified exclusively in ‘Merlot’. During the bottle storage stage, ‘Merlot’ wine did not contain free-form heptyl acetate, ethyl isobutyrate and methionol. Additionally, 14 bound aroma compounds were detected, and their component types did not significantly vary between ‘Marselan’ and ‘Merlot’.

**Figure 2:**
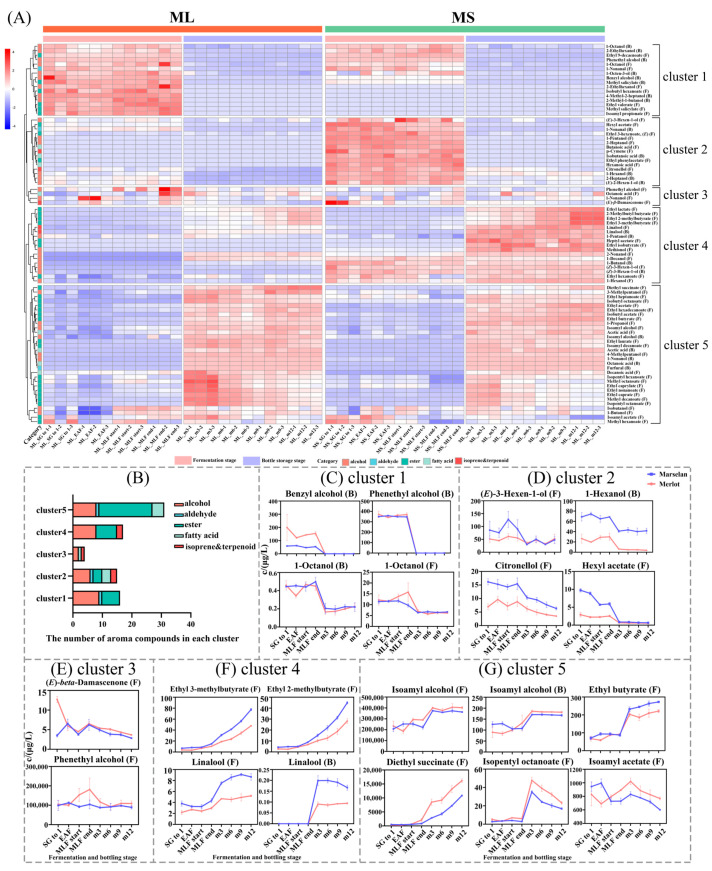
Changes in free−form and glycosidically bound aroma compounds during the fermentation and one-year bottling stages. (**A**) clustered heat map analysis; (**B**) stacked histogram of the number of aroma compounds contained in each cluster; (**C**–**G**) The changes of the aroma compounds with typical variation trend in each cluster. In the heat map, the letter F after the compound name represents the free form, and the letter B represents the bound form.

Figure 2 shows the results of the heat map cluster for free and bound aroma compounds in ‘Merlot’ and ‘Marselan’ wines. The compounds were grouped into five clusters (Figure 2), and Figure 2B summarizes the types and quantities of aroma components in each cluster.

During the alcoholic and malolactic fermentation stages, the aroma compounds in cluster 1 had a high concentration. However, their concentrations decreased significantly from the beginning of bottle storage and remained stable thereafter. These compounds were primarily bound-form alcohols, as shown in Figure 2C. During the fermentation, ‘Merlot’ wine had a higher concentration of bound-form benzyl alcohol compared to ‘Marselan’ wine. However, within the first 3 months of bottle storage, the concentration of bound-form benzyl alcohol decreased rapidly to below the detection limit. The variation trends of both free-form and bound-form 1-octanol were very similar, indicating that the two compounds do not undergo any mutual transformation.

Cluster 2 showed a decrease in concentration during the alcohol fermentation stage, followed by a slight increase during the malolactic fermentation. Afterwards, the compounds dropped significantly to a lower level in the first 3 months of bottle storage. The compounds in cluster 2 were dominated by C5–C7 alcohols and acids, as well as ethyl 3-hexenoate and ethyl phenylacetate. Roughly one-third of the compounds were exclusively present in the fermentation stage of ‘Marselan’ wine, and were not detectable in the ‘Merlot’ wine. The two varieties showed similar declining rates for the aroma compounds detected simultaneously, with only a difference in concentration, such as free-form citronellol and 1-hexanol (Figure 2D).

Aroma compounds in cluster 3 exhibited minimal fluctuation during the fermentation and bottle storage stages, and their concentrations did not differ between the two varieties. This cluster included free (*E*)-*β*-damascenone. The concentration had a small fluctuation during fermentation, and decreased gradually during one-year bottle storage. The concentration of free-form phenethyl alcohol in ‘Marselan’ and ‘Merlot’ wines increased from 1.2 mg/L and 1.4 mg/L before alcohol fermentation (BAF) to about 100 mg/L during alcohol fermentation. This accumulation occurred rapidly in the early stage of fermentation and far exceeded the component’s sensory threshold (14 mg/L) (Figure 2E).

Both cluster 4 and cluster 5 exhibited an overall increasing trend in aroma compounds during the fermentation and bottle storage stages. Cluster 4 continued to accumulate, and reached its peak at the end of bottle storage. The accumulation rate showed variability between the varieties. Using free-form ethyl 3-methylbutyrate and ethyl 2-methylbutyrate as an example, it was found that their accumulation rates were higher in ‘Marselan’ wine than in ‘Merlot’ wine. This difference was more pronounced during the bottle storage stage. Cluster 4 included both free-form and bound-form linalool, as well as ethyl-3-methylbutyrate and ethyl-2-methylbutyrate. During bottle storage, the concentrations of the four compounds were significantly higher in ‘Marselan’ wine than in ‘Merlot’ wine. The differences between the two expanded as the bottle storage time increased (Figure 2F).

Cluster 5 consisted of 31 aroma compounds, including 17 esters, which had higher concentrations in during the bottle storage stage than during the fermentation stage. Most compounds remained stable during one-year bottle storage, and some showed a slow increase in concentration, such as ethyl butyrate, ethyl acetate, isobutyl acetate, and isobutyl octanoate. In contrast, isopentyl octanoate and isoamyl acetate showed a decreasing trend. There were no significant differences in the concentrations of the esters between the ‘Marselan’ and ‘Merlot’ wines.

### 2.4. Aroma Characteristics of ‘Merlot’ and ‘Marselan’ Wines Based on Odor Activity Value

**Figure 3:**
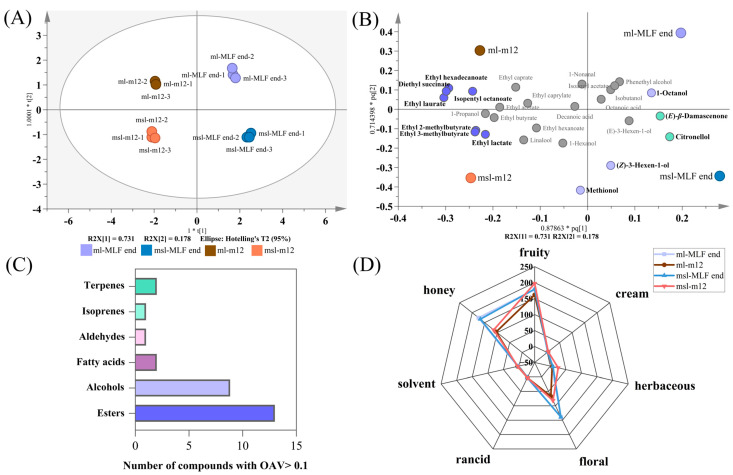
Comparison of aroma profiles based on aroma compounds with OAV > 0.1 at the end of malolactic fermentation and 12-month bottle storage. (**A**) orthogonal partial least squares-discriminant analysis (OPLS−DA) model; (**B**) loading patterns of ‘Marselan’ and ‘Merlot’ wines; (**C**) number of aroma compounds with OAV > 0.1; (**D**) radar chart based on the aroma descriptors of aroma compounds with OAV > 0.1.

To investigate the potential impact of leaching and evolution of aroma compounds on odor characteristics during the vinification process of ‘Marselan’ and ‘Merlot’ dry red wines, we calculated the odor activity value (OAV) of the detected free-form aroma compounds in the wines. It should be noted that the OAV is calculated exclusively for the compounds with absolute quantification. The results are presented in Table S3. In general, aroma compounds with an OAV > 1 are considered to directly contribute to the sensory odor of the wine. The aroma compounds with OVA > 0.1 in wine samples at the end of malolactic fermentation (MLF end) and after 12 months of bottle storage (m12) were selected for OPLS-DA analysis due to the consideration of their interactions (Figure 3A,B).

The analysis revealed 28 aroma compounds with OAV > 0.1. These compounds included eight alcohols (including three C6/C9 compounds, and five higher alcohols), 14 esters (including two acetates, 10 ethyl esters, and two other esters), one aldehyde, two volatile fatty acids, two terpenes, and (*E*)-*β*-damascenone (Figure 3C). The OPLS-DA model (R2X = 0.731, R2Y = 0.178) demonstrated high confidence (Figure 3A). The samples of ‘MLF end’ and ‘m12’ were distinctly separated by the positive and negative halves of the x-axis, while the samples of ‘Merlot’ and ‘Marselan’ were well separated by the positive and negative halves of the y-axis.

Figure 3B shows the loading plot, highlighting the aroma compounds with variance importance (VIP) > 1. Specifically, 1-octanol, citronellol, (*Z*)-3-hexen-1-ol, methionol and (*E*)-*β*-damascenone were distributed on the positive x-axis, indicating their higher abundance in wines at the end of malolactic fermentation compared to those after one-year bottle storage. The OAV values of (*E*)-*β*-damascenone reached 124.6 and 130.4 in the ‘Merlot’ and ‘Marselan’ wines, respectively, at the end of malolactic fermentation (Table S3). These values were approximately twice as high as those found in bottles stored for 12 months. Conversely, isopentyl octanoate, and most ethyl esters were more abundant in the wines after one year of bottle storage, as evidenced by their location on the negative x-axis. These esters have fruity odors, such as apple, banana and lemon. However, their sensory contribution to the aroma of wine is generally lower than that of (*E*)-*β*-damascenone because the compounds have higher sensory threshold values (Table S3). In this study, the chemical characterization of the aroma of ‘Marselan’ wine included linalool, (*E*)-*β*-damascenone, and ethyl esters of C2–C6, while the aroma of the ‘Merlot’ wine was characterized by diethyl succinate, and ethyl or isoamyl esters of C8–C12 (ethyl hexadecanoate, ethyl laurate, ethyl caprate and ethyl caprylate).

Figure 3D displays a radar chart of the odor descriptors based on the compounds with OVA > 0.1. The intensity of floral and honey-like aromas was more pronounced at the ‘MLF end’ stage in comparison to the ‘m12’ stage, with little difference in the other aromas between the two stages. It is proposed that the discrepancy in the strength of floral and honey-like aromas between the two stages was predominantly attributable to the concentration of (*E*)-*β*-damascenone (Figure 3B). The fruity aroma of the wine remained consistent throughout the bottle storage period. Ethyl esters are the primary contributors to fruit aroma, and the majority of their OAVs were found to be greater than 1, indicating their significant impact on the sensory characteristics of the wine. It is postulated that an increase in ethyl ester concentration may serve to prevent the decline of the wine’s fruity aroma during bottle storage.

## 3. Discussion

The study suggests that the effect of cold soak on aroma compounds is dependent on the grape variety, which is consistent with the previous research [23,24], and the leaching rates are affected by the levels of aroma compounds in grape berries. Generally, C6 alcohols, terpenes and norisoprenoids were the main compounds that caused differences in the berries of ‘Marselan’ and ‘Merlot’ grapes. The concentrations of terpenes and norisoprenoids increased during the maceration stage, but their leaching rate was positively correlated with their concentrations in the grapes. Similar patterns were observed for some C6 compounds, such as glycosidically bound (*E*)-2-hexen-1-ol and 1-hexanol, as well as free-form (*Z*)-3-hexen-1-ol. However, other C6 compounds showed continuous increasing or decreasing trends and did not differ between the varieties.

During the fermentation and bottle storage stages of dry red wine, the aroma changes mainly occur in esters. Yeast alcohol fermentation produces a large number of aroma compounds, mainly being acetates and straight-chain acid ethyl esters. Malolactic fermentation mainly produces branched acid ethyl esters, such as ethyl 2-methylbutyrate, ethyl 3-methylbutyrate, heptyl acetate and others. The compounds exhibited an increasing trend, which is consistent with the findings of previous reports [25,26]. Citronellol and linalool were the primary monoterpenoids detected in the two stages. Citronellol, an essential monoterpenoid compound in wine grapes, has a delightful aroma of rose and citrus. Due to its low sensory threshold, citronellol significantly impacts the aroma profile of wine [3]. During the fermentation and bottle storage, the concentration of citronellol continued to decrease, which was opposite to that of linalool, another monoterpenoid. Research has shown that terpenoids undergo molecular arrangement during wine aging, and there is a transformation relationship between citronellol, geraniol, and linalool. This rearrangement is biased towards the production of linalool [27,28]. Therefore, it is believed that this result is due to the chemical changes that occur during the winemaking, specifically the conversion of citronellol to geraniol and then to linalool. This conversion leads to a reduction in citronellol and an increase in linalool.

The bottle storage is a period of wine ripening during which many chemical reactions occur. This study identified two trends in the evolution of aroma compounds during this stage. Firstly, the concentrations of aroma compounds increased during the first three months of bottle storage, and subsequently decreased. The phenomenon described in this text is similar to one observed by Liu et al. [29]. They found that the concentrations of most esters, alcohols, and acids in the ‘Cabernet Sauvignon’ dry red wines increased during the first 3–9 months of the bottle storage, and then declined. The first evolutionary trend contained a large amount of linear long-chain fatty acid ethyl esters, such as ethyl nonanoate and ethyl heptanoate. The decrease in these esters led to a weakening of fruity and floral flavors in the wines [30]. Research has shown that the concentration of dissolved oxygen in wine during bottle storage is the highest at the beginning of bottle storage and then drops sharply within three months [31]. This process leads to the acid-catalyzed hydrolysis of fatty acid ethyl esters, resulting in the formation of acetic acid esters [31,32]. This also explained the rapid decline in the concentrations of fatty acid ethyl esters in the initial stage of bottle storage, and the increase in concentrations of some acetic esters (isobutyl acetate, heptyl acetate, isoamyl acetate, etc.).

Secondly, the concentration of aroma compounds continued to increase over time, with a majority of the included esters being short-chain fatty acid ethyl esters, such as ethyl 2-methylbutyrate, ethyl 3-methylbutyrate, and ethyl butyrate. Additionally, a significant portion of the short-chain fatty acid ethyl esters were branched-chain fatty acid ethyl esters. Research has demonstrated that branched fatty acid ethyl esters are more stable than straight-chain fatty acid ethyl esters and even increase during the aging process [33]. It is believed that the corresponding acids are conversed into branched-chain fatty acid ethyl esters under acid-ester equilibrium, which explains the continuous accumulation of branched-chain fatty acid ethyl esters [34]. Therefore, it can be hypothesized that the effect of bottle storage on fatty acid ethyl esters varied depending on the length of the carbon chain and structural difference. Some aroma compounds, including free ethyl 2-methylbutyrate, accumulated continuously, and their accumulation rate was higher in ‘Marselan’ than in ‘Merlot’. Studies have found that ethyl 2-methylbutyrate has a low boiling point and low log*P* value. The release of esters with these characteristics varies with the level of polyphenol content in wine. At low polyphenol content, there is a general retention phenomenon, while at high polyphenol content, there is a tendency of salting-out effects [35]. The experiments conducted by Juan et al. [36] found that the release of ethyl 2-methylbutyrate was found to be facilitated by an upward trend in red wines, likely due to their high total polyphenol content (TPC). In research of Ling et al., they also suggested that the addition of polyphenols during red wine fermentation can increase the content of various esters, including ethyl 3-methylbutyrate [37]. Therefore, it was believed that the difference in accumulation rate in this experiment was caused by the difference in total polyphenol content (TPC) between “Marselan” and “Merlot”. Previous research has shown that the flavonoid content in “Marselan” is significantly higher than that of “Merlot” [21], which may have led to an enhanced growth rate of aroma compounds in “Marselan”.

The study investigated the evolution of characteristic aromas in dry red wines and their sensory impacts. The dominant aroma difference between the end of the malolactic fermentation and the 12-month bottle samples was proposed to be caused by the (*E*)-*β*-damascenone from the grape berries. The difference in its contents resulted in the lack of floral and honeyed aromas in the dry red wines bottled for 12 months. However, the accumulation of ethyl esters during the bottle-storage stage enriched the fruity aroma of the end-of-bottle-storage samples to some extent. Overall, although there was a significant difference in (*E*)-*β*-damascenone detected in the berries was significantly different between the two varieties, this difference decreased as fermentation and bottle storage progressed. The winemaking process has a greater impact on the aromas of dry red wine compared to the variety.

## 4. Materials and Methods

### 4.1. Winemaking and Sampling

A brief description of sample collection and winemaking can be found here for the reader’s convenience. Details are available in our recently published article [21].

#### 4.1.1. Collection of Grape Berries Samples

Berry samples of Marselan (*Vitis vinifera* L. Marselan) and Merlot (*Vitis vinifera* L. Merlot) were collected from the vineyard of Chateau Yuanshi in Ningxia (106.12° E, 38.28° N). The soluble solids of the berries at harvest were 26.2 ± 0.1 Brix and 25.7 ± 0.1 Brix, respectively, while the titratable acids were 4.70 ± 0.01 g/L and 6.20 ± 0.02 g/L. To ensure the representativeness of the measured fruit aroma compounds, we selected three plots from the ‘Marselan’ and ‘Merlot’ vineyards as three biological replicates. Each biological replicate consisted of 15 vines with similar growth, and we randomly collected 200 grape berries. After picking, the berries were transported to the winery’s testing room within one hour, frozen in liquid nitrogen and refrigerated at −80 °C for subsequent analysis. For the process of winemaking, the berries were packed into large plastic containers and transported to the winery by car.

#### 4.1.2. Winemaking and Sampling

Zhang et al. [21] described the winemaking of ‘Marselan’ and ‘Merlot’ single-variety wines. Each monovarietal wine was fermented in one 20-kiloliter (kL) fully automated thermostatic fermenters along with 4.0 g/hL of K_2_S_2_O_5_ and 2.0 g/hL of pectinase. In consideration of the distinctive characteristics of the grapes from this region, namely their low acidity and high sugar content, 150 g/hL and 110 g/hL of tartaric acid were incorporated into the musts of ‘Merlot’ and ‘Marselan’, respectively, before fermentation. This was done in accordance with the local production guidelines. The objective of the addition was to enhance the stability of the wine color and to improve its mouthfeel, while compensating for the low titratable acid. Cold maceration was then carried out for 3 days at around 10 °C. At a temperature of 10 °C, 14.0 g/hL of pre-activated OKAY yeast (LALLEMAND, Niagara-on-the-Lake, ON, Canada) and XPURE yeast (Laffort, Bordeaux, France) were added to ‘Merlot’ and ‘Marselan’ fermenters, respectively, to initiate alcohol fermentation. The temperature was controlled at 20–25 °C. Additionally, the wine was punched every 8 h for 20 min from the beginning of cold maceration until the end of alcohol fermentation. Alcohol fermentation was considered complete when the specific gravity dropped to around 0.993 and residual sugar was less than 4 g/L. The residue was then separated by racking. The free-run wine was loaded into a 10 kL stainless steel fermenter, and 5 mg/L VP41 lactobacillus (Enartis, Niagara-on-the-Lake, ON, Canada) was added to initiate malolactic fermentation, with a controlled temperature at 18–20 °C. Malolactic fermentation process was monitored by using the FOSS WineScan instrument (Foss Electric, Hillerød, Denmark) to examine the changes in malic acid concentration. The fermentation was deemed to be complete when the concentration of malic acid dropped below 0.3 mg/L in accordance with local regulations pertaining to wine making technology.

The residual sugar, total acid, volatile acid (VA) and pH of the wines were measured using a fast scanning infrared Fourier transform spectrometer from Foss Winescan. The results indicate that the alcohol content of ‘Marselan’ and ‘Merlot’ wines was 14.88 ± 0.04% (*v*/*v*) and 14.31 ± 0.01% (*v*/*v*), respectively, while the residual sugar was 4.87 ± 0.31 g/L and 3.63 ± 0.29 g/L, respectively. According to the study, the total acid levels were 7.80 ± 0.01 g/L and 6.80 ± 0.01 g/L, while the volatile acid levels were 0.67 ± 0.02 g/L and 0.67 ± 0.01 g/L, respectively. The pH were 3.28 ± 0.01 and 3.54 ± 0.01, respectively. The titratable acid content of the wines was found to exceed that of the grape juice, primarily due to the addition of tartaric acid prior to alcohol fermentation, as previously mentioned [21]. The wine samples were bottled and stored in an underground wine cellar at a temperature of approximately 16 °C with a controlled relative humidity of 70%.

The wine samples were collected at various stages of the winemaking process, including must, before cold maceration (BC), after cold maceration (EC), before heating (BH), before alcohol fermentation (BAF), specific gravity down to 1.00 (SG to 1), the end of alcohol fermentation (EAF), the beginning of malolactic fermentation (MLF start), and the end of malolactic fermentation (MLF end). Additionally, samples were taken during bottle storage stages at 3 months (3 m), 6 months (6 m), 9 months (9 m), and 12 months (12 m). For each sampling point, three bottles of 350 mL were taken and stored separately in a refrigerator at −40 °C.

### 4.2. Determination of Free and Bound Aroma Compounds in Grape Berries

#### 4.2.1. Extraction of Aroma Compounds in Berries

The laboratory’s previous method was adopted with a few modifications [38]. All samples were analyzed in triplicate.

For pre-treatment, ‘Marselan’ and ‘Merlot’ fruit samples (70–100 g) were destemmed and deseeded under the protection of liquid nitrogen, with 0.5 g of D-gluconolactone and 2 g of polyvinylpyrrolidone (PVPP) (both purchased from Sigma (St. Louis, MO, USA)). Then, the grape berries were ground into a powder and placed in 50 mL centrifuge tubes. The tubes were then stored at 4 °C for 8 h before being centrifuged at 8000 rpm for 15 min at 4 °C. The resulting supernatant was collected for further analysis.

The extraction of free aroma compounds followed. The 5 mL of clarified grape juice was transferred to a 20 mL vial with a polytetrachloroethylene (PTFE)-silicon diaphragm. Then, 1 g of NaCl and 10 μL of internal standard (1.0018 g/L 4-methyl-2-pentanol) were added, and the bottle cap was tightened for sampling.

The extraction of glycosidically bound aroma compounds followed. The Cleanert PEP-SPE solid phase extraction column, made of polystyrene/divinylbenzene and 150 mg/6 mL, was activated with 10 mL of methanol and 10 mL of water, respectively. Next, 2 mL of clarified grape juice was added to the column. Afterwards, 5 mL of water and 5 mL of dichloromethane were used to remove low-molecular-weight sugars, acids, free-form aroma compounds, and other polar compounds. Finally, glycosidically bound aroma compounds were eluted with 20 mL of methanol. The methanol collection solution was evaporated under reduced pressure in a steam evaporator until dry. Next, the sample was redissolved in 10 mL of citric acid-phosphate buffer (0.2 M, pH 5). Then, 100 μL of glycosidase AR2000 (100 g/L) was added, and the enzymatic hydrolysis was carried out for 16 h at 37 °C. After completing of the enzymatic hydrolysis, the hydrolasate’s pH was adjusted to 3.0 using citric acid. In the end, a 20 mL bottle was used to combine 5 mL of extract solution, 1 g NaCl and 10 μL internal standard (1.0018 g/L 4-methyl-2-pentanol) prior to analysis.

#### 4.2.2. Determination of Aroma Compounds in Grape

The aroma compounds in the grapes were detected using a solid-phase microextraction (SPME)-Agilent 6890 Gas Chromatography (GC)/5975C Mass Spectrometry (MS) (Agilent Technologies, Santa Clara, CA, USA) method that has been optimized in our laboratory [38]. We used a CTC CombiPAL autosampler (CTC Analytics, Zwingen, Switzerland) with a DVB/CAR/PDMS 50/30 μm SPME fiber (Supelco, Bellefonte, PA, USA). The extraction head was activated at 250 °C, and then inserted into the top of the vial to extract the volatile compounds at 40 °C for 30 min. The column chamber was initially held at 50 °C for 1 min and then heated to 220 °C at a rate of 3 °C/min and held for 5 min. The Agilent 19091N-136 HP-INN0Wax column (60.0 m × 0.25 mm × 0.25 μm) was thermally performed at 250 °C for 8 min using high purity helium as the carrier gas with a flow rate of 1.0 mL/min (purity > 99.999%) in a splitless mode. The molecules of the aroma compounds were ionized using the electron ionization (EI) at 70 eV energy. The ion source and mass spectrum interface were maintained at temperatures of 230 °C and 280 °C, respectively. The mass scanning range was *m*/*z* 30~350.

#### 4.2.3. Identification and Quantification of Grape Aroma Compounds

The aroma compounds were identified using the automatic mass spectrum deconvolution qualitative system (AMDIS) for spectral decomposition [38]. The retention time (RI) and signal-to-noise ratio (S/N) of the detected aroma compounds were compared with the mass spectrum information of the compounds in NIST11 to characterize the aroma compounds. Retention index (RI) is used to standardize the retention times of aroma compounds on the chromatographic column, and it was calculated using n-alkanes from C6 to C24 as reference compounds. Subsequently, a quantitative method was established in Data Analysis software to calculate the peak area ratio of each compound to the internal standard. For aroma compounds with standards, absolute quantification was performed by directly substituting into the calibration curves generated from the reference standards. For compounds without standards, relative quantification was performed based on standards with similar numbers of carbon atoms or the same functional groups. The standard solution with a mixed aroma was prepared using simulated grape juice comprising of 7 g/L tartaric acid, 200 g/L glucose, with pH adjusted to 3.3 using a 5 mol/L NaOH solution. The solution was prepared with 12 concentration gradients, and the peak area ratios for each concentration were obtained through sampling. A standard curve was then obtained using the peak area ratio as the x value and the compound concentration as the y value.

### 4.3. Determination of Aroma Compounds in the Free and Bound States of Wine

#### 4.3.1. Extraction of Wine Aroma Compounds

Wine aroma compounds are extracted using the same method as described in Section 4.2.1.

#### 4.3.2. Determination of Wine Aroma Compounds

To determine the aroma compounds in the samples, the SPME-Agilent 7890 GC/5975C MS method was used. The samples were injected in 5:1 split mode. The temperature was increased according to the following procedure: the initial column temperature was set to 50 °C and held for 1 min. It was then raised to 220 °C at a rate of 3 °C/min and held for 3 min. Finally, the temperature was increased to 240 °C at a rate of 10 °C/min and held for 5 min. The remaining method was consistent with those described in Section 4.2.2.

#### 4.3.3. Determination of Free and Bound Aroma Compounds in Wine

To prepare the aroma standard mixed solution, we used a simulated wine solution. We added 2 g/L glucose and 6 g/L tartaric acid to a 13% *v*/*v* aqueous ethanol solution and adjusted the pH of the solution to 3.5 with NaOH. The remaining experimental operations were the same as in Section 4.2.3.

### 4.4. Statistical Analysis

The data were collected by using Microsoft Excel. The line graph was made using Graphpad Prism 9.0 software (GraphPad Software, San Diego, CA, USA). IBM SPSS Statistics 27 (IBM, Armonk, NY, USA) was used to analyze the significant differences between data groups, employing one-way analysis of variance with a significance level of 5% (*p* < 0.05). To identify changes in aroma compound concentrations, heat maps were generated using the ‘pheatmap’ package of R 4.2.2 (RStudio, Boston, MA, USA) to find changes in aroma substances contents. Principal component analysis (PCA) was performed using SIMCA software (version 14.1 from Umetrics, Umea, Sweden).

## 5. Conclusions

This study examines the evolution of free-form and glycosidically bound aroma compounds from grapes to wines of ‘Marselan’ and ‘Merlot’, respectively, and their differences. It is found that most alcohols are quickly extracted during the cold soak stage. Some C6 alcohols, such as (*Z*)-3-hexen-1-ol, as well as linalool and (*E*)-*β*-damascenone show varietal variability in the extraction rate, with a higher leaching rate from the ‘Marselan’ grapes than the ‘Merlot’. The extraction variability in aroma compounds of grape berries is believed to be primarily due to the difference in their concentration levels. Numerous ethyl esters are produced during alcohol fermentation. Among them, branched short-chain fatty acid ethyl esters, such as ethyl 3-methylbutyrate and ethyl 2-methylbutyrate, exhibit stable increase throughout the fermentation and one-year bottle storage. This suggests that this is a crucial quality control point for the retention of fruity flavors in wine. In the context of wine-making, the promotion of the production of these compounds would be beneficial for the purpose of obtaining high-quality fruity aromas. Furthermore, the rate of increase in ‘Marselan’ wine is higher than that of ‘Merlot’ wine, indicating that the high level of phenolic compounds in the ‘Marselan’ wine may cause a salting-out effect on the detectability of aroma compounds. Additionally, the accumulation of this fraction of ethyl esters during bottle storage partially compensates for the reduction in fruity aroma intensity in wines caused by the decline in (*E*)-*β*-damascenone to some extent.

In summary, these findings provide a basis for better understanding of the evolution of aroma profiles, and for achieving a precise regulation of wine aroma and flavor, which has significant production value.

## Data Availability

Data are contained within the article or Supplementary Materials.

## Supplementary Materials

The following supporting information can be downloaded at: https://www.mdpi.com/article/10.3390/molecules29143250/s1, Figure S1: Clustered heat map of free-form and glycosidically bound aroma compounds between the samples before alcoholic fermentation (BAF) and in specific gravity down to 1.00 (SG to 1); Table S1: Changes in the concentration of aroma substances during the maceration stage of wines (μg/L); Table S2: Changes in the concentration of aroma substances during the processes of fermentation and bottle storage of wines (μg/L); Table S3: Concentrations, odor thresholds, odor activity values (OAVs), aroma descriptors and series of volatile aroma compounds at the end of malolactic fermentation (MLF end) and bottling for 12 months (m12). References [39,40,41,42,43,44,45,46,47,48] are cited in the supplementary materials.

## Abbreviations

BC	Before cold maceration
EC	After cold maceration
BH	Before heating
BAF	Before alcoholic fermentation
SG to 1	Specific gravity down to 1.00
EAF	The end of alcoholic fermentation
MLF start	The beginning of malolactic fermentation
MLF end	The end of malolactic fermentation
3m	The bottle storage stages of 3 months
6m	The bottle storage stages of 6 months
9m	The bottle storage stages of 9 months
12m	The bottle storage stages of 12 months
MS	Marselan
ML	Merlot
OVA	The odor activity value
VIP	Variance importance
