# Supplementary material for: Evolution of Aroma Profiles in Vitis vinifera L. Marselan and Merlot from Grapes to Wines and Difference between Varieties
Source: Molecules. 2024 Jul 9;29(14):3250. doi: 10.3390/molecules29143250 (PMC11279373; doi:10.3390/molecules29143250)
Supplement: Supplementary file 1 [file molecules-29-03250-s001.zip › molecules-3010399-supplementary/Supplementary Material/molecules-supplementary figure.pdf]

Supplementary materials

Evolution of Aroma Profiles in *Vitis Vinifera* L. Marselan and Merlot from Grapes to Wines and Difference between Varieties

Yi-lin Ge [1,2], Nong-yu Xia [1,2], Ya-chen Wang [1,2], Hua-lin Zhang [1,2], Wei-ming Yang [3], Changqing Duan [1,2] and Qiu-Hong Pan [1,2,*]

1 Center for Viticulture and Enology, College of Food Science and Nutritional Engineering, China Agricul-tural University, Beijing 100083, China 2 Key Laboratory of Viticulture and Enology, Ministry of Agriculture and Rural Affairs, Beijing 100083, China

3 Chateau Zhihui Yuanshi Co. Ltd., 750026 Yinchuan, Ningxia, China *
Author to whom correspondence should be addressed.

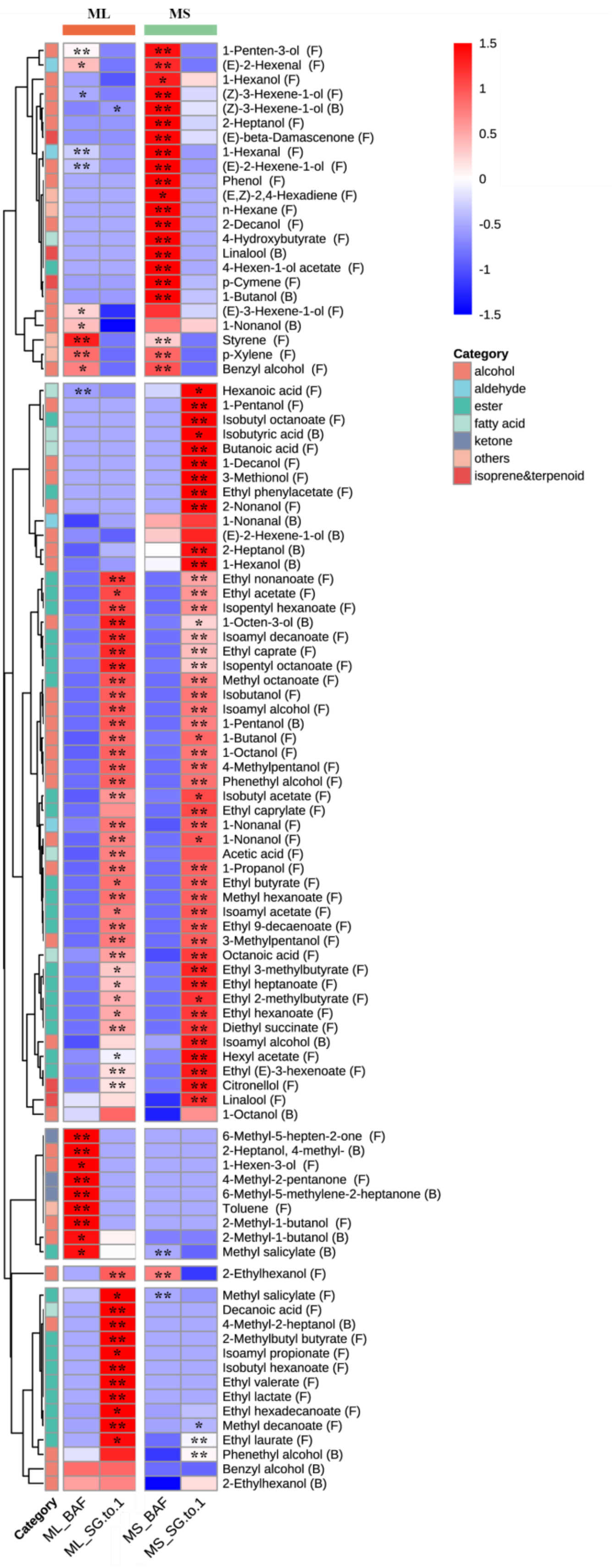

Figure S1. Clustered heat map of free-form and glycosidically-bound aroma compounds between the samples before alcoholic fermentation (BAF) and in specific gravity down to 1.00 (SG to 1)
